# 5-AZA Upregulates SOCS3 and PTPN6/SHP1, Inhibiting STAT3 and Potentiating the Effects of AG490 against Primary Effusion Lymphoma Cells

**DOI:** 10.3390/cimb46030156

**Published:** 2024-03-14

**Authors:** Michele Di Crosta, Andrea Arena, Rossella Benedetti, Maria Saveria Gilardini Montani, Mara Cirone

**Affiliations:** Department of Experimental Medicine, Sapienza University of Rome, Viale Regina Elena 324, 00161 Rome, Italy; michele.dicrosta@uniroma1.it (M.D.C.); a.arena@uniroma1.it (A.A.); rossella.benedetti@uniroma1.it (R.B.)

**Keywords:** STAT3, PEL, 5-Azacytidine, AG490, valemetostat

## Abstract

Epigenetic modifications, including aberrant DNA methylation occurring at the promoters of oncogenes and oncosuppressor genes and histone modifications, can contribute to carcinogenesis. Aberrant methylation mediated by histone methylatransferases, alongside histones, can affect methylation of proteins involved in the regulation of pro-survival pathways such as JAK/STAT and contribute to their activation. In this study, we used DNA or histone demethylating agents, 5-Azacytidine (5-AZA) or DS-3201 (valemetostat), respectively, to treat primary effusion lymphoma (PEL) cells, alone or in combination with AG490, a Signal transducer and activator of transcription 3 (STAT3) inhibitor. Cell viability was investigated by trypan blue assay and FACS analysis. The molecular changes induced by 5-AZA and/or AG490 treatments were investigated by Western blot analysis, while cytokine release by PEL cells treated by these drugs was evaluated by Luminex. Statistical analyses were performed with Graphpad Prism^®^ software (version 9) and analyzed by Student’s *t* test or a nonparametric one-way ANOVA test. The results obtained in this study suggest that 5-AZA upregulated molecules that inhibit STAT3 tyrosine phosphorylation, namely Suppressor of Cytokine Signaling 3 (SOCS3) and tyrosine–protein phosphatase non-receptor type (PTPN) 6/Src homology region 2 domain-containing phosphatase-1 (SHP-1), reducing STAT3 activation and downregulating several STAT3 pro-survival targets in PEL cells. As this lymphoma is highly dependent on the constitutive activation of STAT3, 5-AZA impaired PEL cell survival, and when used in combination with AG490 JAK2/STAT3 inhibitor, it potentiated its cytotoxic effect. Differently from 5-AZA, the inhibition of the EZH1/2 histone methyltransferase by DS-3201, reported to contribute to STAT3 activation in other cancers, slightly affected STAT3 phosphorylation or survival in PEL cells, either alone or in combination with AG490. This study suggests that 5-AZA, by upregulating the expression level of SOCS3 and PTPN6/SHP1, reduced STAT3 activation and improved the outcome of treatment targeting this transcription factor in PEL cells.

## 1. Introduction

Signal transducer and activator of transcription (STAT3) plays a key role in all steps of carcinogenesis, from cancer onset to cancer survival and progression [1]. Although rarely mutated, this pathway results in several constitutively activated solid and hematological cancers, including primary effusion lymphoma (PEL) [2]. This is a rare B cell lymphoma associated with Kaposi sarcoma-associated herpesvirus (KSHV) and in several cases also with Epstein–Barr virus (EBV).

The current therapies used against this lymphoma include cyclophosphamide, doxorubicin, vincristine, and prednisone (CHOP) chemotherapy, and the proteasome inhibitor Bortezomib, although none of these can provide complete remission from disease [3]. Besides being activated by autocrine signaling triggered by the released IL-6, STAT3 can be phosphorylated in PEL cells, both at Y705 tyrosine and S727 serine, by the expression of viral proteins, including the KSHV-encoded IL-6 (v-IL-6) that shares about 25% homology with cellular IL-6 [4]. Previous studies have shown that the inhibition of STAT3 Y705 tyrosine phosphorylation, mainly mediated by Janus activating kinases (JAK) 2, may be an effective strategy for reducing the survival of PEL cells [5,6]. Notably, STAT3 engages in crosstalk with other pro-survival pathways, e.g., nuclear factor erythroid 2–related factor 2 (NRF2) [7] and mutant p53 [8,9], to further promote tumorigenesis; on the other hand, it may negatively influence the activation of wtp53 in PEL cells [10]. Other than phosphorylation, STAT3 may undergo other post-translational modifications that affect its activity, and among those, it may be acetylated [11] or methylated [12,13]. Regarding the latter modification, it has been reported that STAT3 can be dimethylated or trimethylated at K49 and K180 by EZH2 (an enhancer of the zeste homolog) 2 [12,14] and dimethylated on K140 by the histone methyltransferase SET9 (SET domain-containing lysine methyltransferase 9) [13]. While the methylation mediated by SET9 occurs when STAT3 is bound to the promoter and positively regulates only a subset of target genes such as suppressor of cytokine signaling (SOCS) 3, the methylation induced by EZH2 can induce a broader STAT3 activation, contributing to the oncogenic properties of this transcription factor. More recently, it has been shown that STAT3 may also be asymmetrically dimethylated by histone arginine N-methyltransferase 6 (PRMT6) at arginine 729 (STAT3 R729me2a), increasing the capacity to induce metastasis in breast cancer [15].

DNA methylation of promoters of genes negatively regulating STAT3, such as SOCS3, can also influence STAT3 activity. Interestingly, SOCS3, as mentioned above, is also a target of STAT3, which limits the threshold of its activation. It belongs to a family that comprises eight members, having a central SH2 domain that inhibits the JAK/STAT pathway [16]. Aberrant methylation of the CpG of SOCS3 promoters has been reported to result in increased phosphorylation and activation of STAT3 in cancers such as lung cancer and hepatocellular carcinoma [16,17]. 

Among other molecules that negatively regulate STAT3, there is tyrosine–protein phosphatase non-receptor type (PTPN) 6, also known as Src homology region 2 domain-containing phosphatase-1 (SHP-1) [18]. Promoter methylation of PTPN6 occurs in acute myeloid leukemia (AML) cells; indeed, treatment with 5-Azacytidine (5-AZA), an inhibitor of the DNA methyltransferase (DNMT) 1, upregulated PTPN6, thereby resulting in STAT3 inhibition. Due to its inhibitory activity on STAT3, the gene encoding for PTPN6 can be considered a tumor suppressor gene in AML [19]. Promoter methylation of PTPN6 leads to a very low level of expression of this protein, representing a tumor suppressor candidate in other hematological cancers, such as in multiple myeloma (MM) [20]. Interestingly, STAT3 may contribute to the epigenetic silencing of PTPN6, together with DNMT1 [21], to sustain its own activation. 

Given the pro-survival role of STAT3 in PEL cells and given that the activity of this transcription factor may be influenced by its methylation or that of promoters of genes encoding for proteins inhibiting it, in the present study, we explored the impact of DNA- or histone-demethylating agents on STAT3 activation. To this end, 5-AZA or valemetostat EZH1/2 inhibitors were used, and as well as STAT3 tyrosine phosphorylation, the expression of pro-survival molecules regulated by this transcription factor were investigated. Moreover, we evaluated whether these demethylating agents could reduce cell survival or improve the cytotoxic effect of drugs targeting STAT3, such as AG490, against PEL cells.

## 2. Materials and Methods

### 2.1. Cell Cultures and Treatments

BC3 (ATCC, CRL-2277) and BCBL-1 (kindly supplied by Prof. P. Monini, National AIDS center, Istituto Superiore di Sanità, Rome, Italy) are human B cell lines derived from primary effusion lymphoma (PEL). Cells were grown in RPMI 1640 medium (Sigma-Aldrich, Burlington, MA, USA) supplemented with 10% fetal bovine serum (FBS) (Sigma-Aldrich, Burlington, MA, USA), L-glutamine (2 mM) (Aurogene, Rome, Italy), and streptomycin/penicillin (100 μg/mL) (Aurogene, Rome, Italy) (complete medium) at 37 °C in a 5% CO_2_ humified incubator. Cells were seeded into 6-well plates at a density of 4 × 105/well in 2 mL of complete medium and were treated for 48 h with 5-Azacitidine (5-AZA) (20 nM or 1 μM) (Selleckchem, Mathias Brueggen Strasse 160, Cologne Germany) added every 24 h or with valemetostat (DS-3201) (1 μM or 2 μΜ) (Selleckchem, Mathias Brueggen Strasse 160, Cologne, Germany). In some experiments, BC3 and BCBL1 were pre-treated or not with 5-AZA (20 nM) (Selleckchem, Mathias Brueggen Strasse 160, Cologne, Germany) or valemetostat (DS-3201) (1 μM) (Selleckchem, Mathias Brueggen Strasse 160, Cologne, Germany) for 24 h and then treated for other 24 h with or without AG490 (100 μM) (Calbiochem, San Diego, CA, USA; #658411). 5-AZA was added every 24 h. DMSO-treated cells were used as control.

### 2.2. Trypan Blue Assay

After treatment, a trypan blue (Sigma-Aldrich, Burlington, MA, USA, #72571) dye exclusion assay was performed to determine the number of viable cells. Unstained cells (live cells) were counted by light microscopy using a Neubauer hemocytometer. The experiments were performed in triplicate and repeated at least three times.

### 2.3. Sub G1 Analysis

For sub G1 analysis, the DNA content of BC3 and BCBL-1 cells, treated as reported above, was performed using the method of Propidium Iodide (P.I.) (Sigma Aldrich, St. Louis, MO, USA; #P4170) staining and flow cytometry analysis.

### 2.4. Western Blot Analysis

After treatment, the cells were harvested, washed with phosphate-buffered saline (PBS; Thermofisher Scientific, Waltham, MA, USA, #18912014), and centrifuged at 1200 rpm (revolutions per minute) for 5 min at room temperature (RT), and cell pellets were lysed in RIPA buffer (150 mM NaCl, 1% NP-40, 50 mM Tris-HCl (pH 8), 0.5% deoxycholic acid, 0.1% SDS, protease, and phosphatase inhibitors). The protein concentration was measured using a Quick Start Bovine Serum Albumin (BSA) assay (Bio-Rad, Hercules, CA, USA), and 12 μg of protein was denatured in loading buffer by heating for 10 min at 70 °C and subjected to electrophoresis on 4–12% NuPage Bis-Tris gels (Life Technologies, Carlsbad, CA, USA) according to the manufacturer’s instructions. The gels were transferred to nitrocellulose membranes (Bio-Rad, Hercules, CA, USA) for 1 h in tris-glycine buffer, and then the membranes were stained with Ponceau S staining solution (SERVA Electrophoresis GmbH, Heidelberg, Germany, #33427.01) to verify protein transfer. After 1 h of blocking in 1× PBS-0.1% Tween20 3% BSA (Applichem, GMBH, Darmstadt, Germany, #A1391,0100), the membranes were incubated with specific primary antibodies overnight at 4 °C. The blots were subsequently washed three times with 1× PBS 0.1% Tween 20 and incubated with the suitable HRP-conjugated secondary antibody for 30 min at RT. After three washes, the membranes were subjected to ECL (Advansta, San Jose, CA, USA, #12045-D20).

### 2.5. Antibodies

To evaluate protein expression on Western blot membranes, the following antibodies were used: mouse monoclonal anti-pSTAT3 TYR (1:500) (pY705, BD Biosciences, Franklin Lakes, NJ, USA, #612356), mouse monoclonal anti-STAT3 (1:500) (BD Biosciences, #6101289), rabbit polyclonal anti-DNMT1 (1:1000) (Proteintech Europe, Manchester, UK, #24206-1-AP), rabbit polyclonal anti-PTPN6 (1:250) (Proteintech Europe, Manchester, UK, #24546-1-AP), mouse monoclonal anti-Cyclin D1 (1:100) (Santa Cruz Bio-technology, #sc-8396), rabbit polyclonal anti-c-MYC (1:500) (Proteintech Europe, Manchester, UK, #10828-1-AP), rabbit polyclonal anti-SOCS3 (1:500) (Proteintech Europe, Manchester, UK, #14025-1-AP), rabbit polyclonal anti-HSP27 (1:5000) (Proteintech Europe, Manchester, UK, 1#8284-1-AP), and mouse monoclonal anti-JACK2 (1:100) (Santa Cruz Biotechnology, Dallas, TX, USA, #sc-390539); mouse mono-clonal anti-GAPDH (1:10,000) (Santa Cruz Biotechnology, Dallas, TX, USA, sc-137179) was used as loading control. The peroxidase goat anti-mouse IgG (1:20,000) (Jackson ImmunoResearch, West Grove, PA, USA, #115-035-062) and goat anti-rabbit IgG-HRP (1:30,000) (Advansta, San Josè, CA, USA, #R-05072-500) were used as secondary antibodies. All the primary and secondary antibodies were diluted in PBS 0.1% Tween20 solution containing 3% BSA.

### 2.6. Chemiluminescent Immunometric Assay

Supernatants from BC3 and BCBL-1 cells treated as above described with a 24 h 5-Azacitidine pre-treatment and with AG490 treatment combined or not with 5-AZA for total 48 h were collected, and Interleukin-6 (IL-6), Interleukin-10 (IL-10), vascular endothelial growth factor (VEGF), and Cathepsin S were measured by Magnetic Luminex assay using a human premixed multi-analyte kit (R&D Systems Bio-Techne, LXSAHM, Minneapolis, MN, USA), following the manufacturer’s instructions.

### 2.7. Densitometric Analysis

The quantification of protein bands was performed by densitometric analysis using Image J software (1.47 version, NIH, Bethesda, MD, USA), which was downloaded from the NIH website (http://imagej.nih.gov, accessed on 10 February 2022).

### 2.8. Statistical Analysis

Results are represented by the mean ± standard deviation (S.D.) of at least three independent experiments, and statistical analyses were performed with Graphpad Prism^®^ software (Version 9.0.0 Graphpad software Inc., La Jolla, CA, USA). Student’s *t* test or a nonparametric one-way ANOVA test was used to demonstrate statistical significance. Difference was considered statistically significant when the *p*-values were * < 0.05; ** < 0.01; *** < 0.001 and **** < 0.0001; ns means not significant.

## 3. Results

### 3.1. 5-AZA Reduces Cell Survival and STAT3 Y705 Tyrosine Phosphorylation and c-Myc, Cyclin D1 and HSP27 Expression Levels While Upregulating SOCS3 and SHP1 in PEL Cells

**Figure 1:**
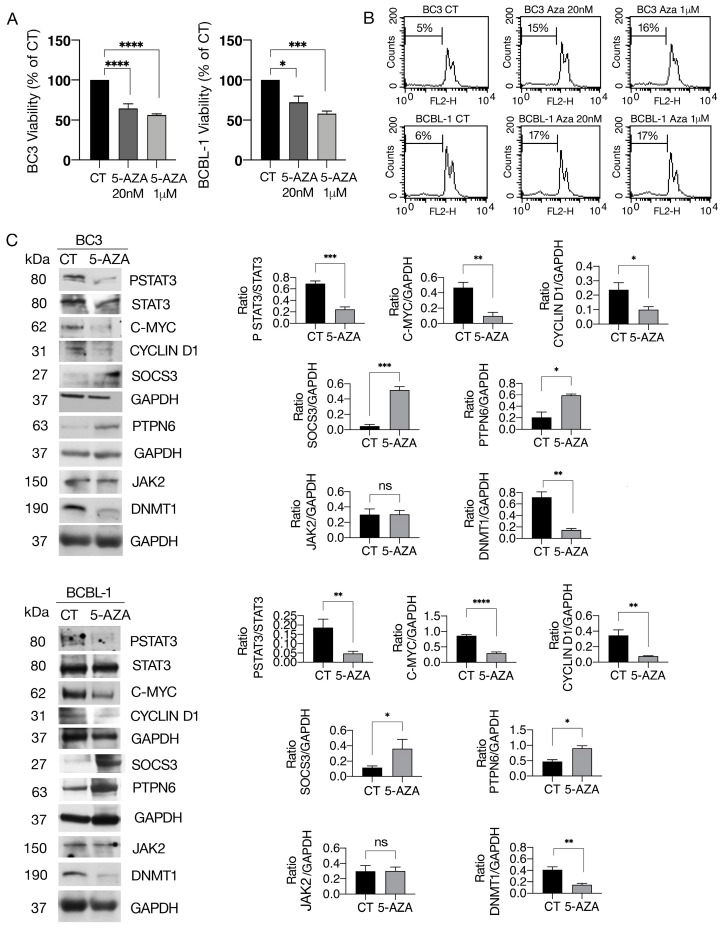
5-AZA induces a cytotoxic effect in PEL cells in correlation with the inhibition of STAT3 and its targets. BC3 and BCBL-1 cell lines were treated with 5-Azacitidine (5-AZA) for 48 h. (**A**) Cell survival was evaluated by trypan blue exclusion assay. The histograms represent the mean of the percentage of cell viability relative to the control plus S.D. (**B**) FACS profiles and percentage of sub-G1 events as evaluated by FACS analysis in BC3 and BCBL-1 cells. One representative experiment out of three is shown. (**C**) Protein expression level of pSTAT3, STAT3, c-MYC, SOCS3, PTPN6, JACK2, and DNMT1, as evaluated by Western blot analysis. GAPDH was used as a loading control, and one representative experiment is shown. The histograms represent the densitometric analysis of specific protein and the appropriate control from three experiments. Data are represented as the mean plus S.D. *p*-value: * < 0.05; ** < 0.01; *** < 0.001; **** < 0.0001 and ns means no significance.

PEL cells were exposed to the DNA de-methylating agent 5-AZA at two different doses (20 nM and 1 µM), and the effect on cell survival was evaluated, as it is known to affect cell viability in other hematological and solid cancers, [22]. As shown in Figure 1A, 5-AZA impaired the survival of BC3 and BCBL-1 PEL cells, although the cytotoxic effect slightly increased with increasing dose. Cell cycle analysis showed that the number of sub-G1 events increased following treatment by 5-AZA, suggesting apoptosis induction (Figure 1B). Given that DNA methylation has been reported to silence the expression of tumor suppressor genes including phosphatases inhibiting STAT3, here, we evaluated if 5-AZA could reduce the tyrosine phosphorylation of STAT3, a pathway constitutively activated and required for PEL cells’ survival. As shown in Figure 1C, STAT3 Y705 phosphorylation was reduced by 5-AZA in both BC3 and BCBL-1 PEL cell lines. The expression levels of pro-survival molecules known to be under the control of STAT3, such as c-Myc [23], playing a key role in sustaining PEL cell proliferation [24], and Cyclin D1 [25], required for cell cycle progression, were investigated. According to the inhibitory effect on STAT3 phosphorylation, 5-AZA reduced the expression level of these molecules in PEL cells (Figure 1C), which could contribute to the reduction in cell PEL survival induced by this DNA-demethylating agent. We then asked if STAT3 de-phosphorylation by 5-AZA could correlate with the upregulation of SOCS3 and/or PTPN6, as the former is a JAK/STAT inhibitor [26] and the latter is a tyrosine phosphatase [27] whose promoter methylation has been shown to contribute to STAT3 activation in other cancers. The results shown in Figure 1C suggest that 5-AZA upregulated both SOCS3 and PTPN6 in PEL cells, an effect that may contribute to STAT3 de-phosphorylation. We finally investigated whether the expression of JAK2, the kinase most involved in STAT3 phosphorylation, could be also affected by 5-AZA and found that it was not influenced by this treatment (Figure 1C). According to previous findings [28], we found that 5-AZA was able to reduce the expression level of DNMT1 in both PEL cell lines.

### 3.2. 5-AZA Potentiates the Anti-Cancer Effects of AG490 Y705 Tyrosine STAT3 Inhibitor

**Figure 2:**
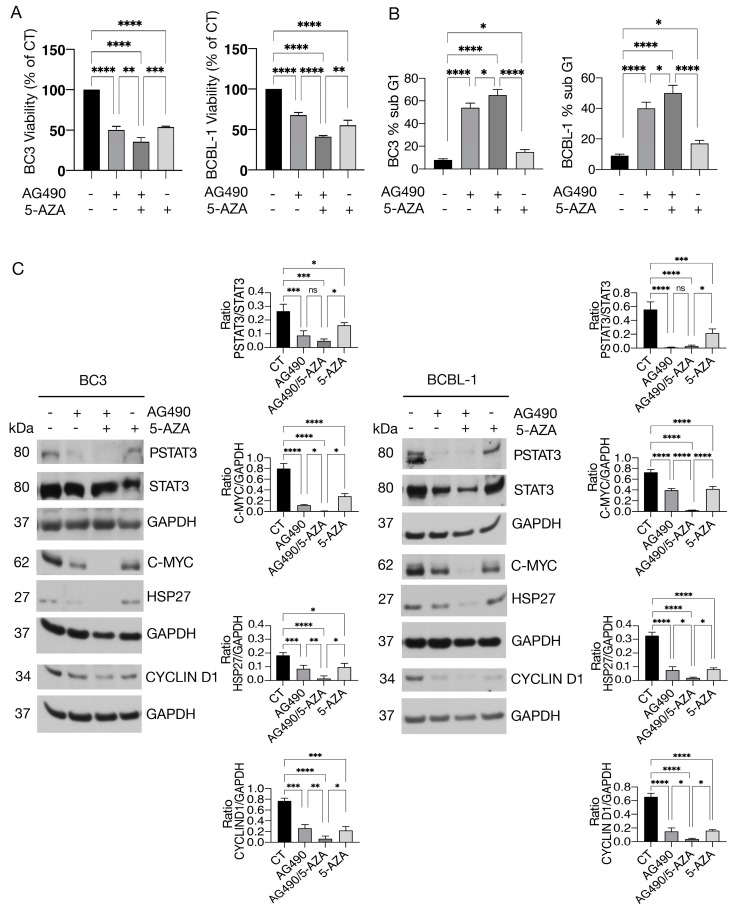
5-AZA further inhibits STAT3 activity in PEL cells treated by AG490. BC3 and BCBL-1 cell lines were treated singly or in combination with 5-Azacitidine (5-AZA) and AG490. (**A**) Cell survival was estimated by trypan blue exclusion assay after treatment with AG490 (100 μM), 5-AZA (20 nM), or a combination of both. The histograms represent the mean of the percentage of cell viability relative to the control plus S.D. (**B**) Sub-G1 events as evaluated via FACS analysis in BC3 and BCBL-1 cells. The histograms represent the mean of the percentage of sub G1 of three independent experiments. (**C**) Protein expression level of pSTAT3, STAT3, c-MYC, HSP27, Cyclin D1, as evaluated by Western blot analysis. GAPDH was used as a loading control, and one representative experiment is shown. The histograms represent the densitometric analysis of specific protein and the appropriate control from three experiments. Data are represented as the mean plus S.D. *p*-value: * < 0.05; ** < 0.01; *** < 0.001; and **** < 0.0001, ns means no significance.

We next investigated whether 5-AZA could potentiate the cytotoxic effect of AG490, an inhibitor of STAT3 Y705 phosphorylation previously reported to reduce PEL cell survival [5]. To this end, BC3 and BCBL-1 cells were pre-treated with 5-AZA (20 nM) and then exposed to AG490 (100 µM) in combination with 5-AZA for additional 24 hours. As shown in Figure 2A,B, a higher cytotoxic effect and an increased number of sub-G1 events were observed following 5-AZA/AG490 combination treatment. Even if the STAT3 dephosphorylation was slightly further reduced by AZA/AG490 combination compared to AG490, a stronger downregulation of c-Myc, Cyclin D1 and HSP27 was observed (Figure 2C). The overexpression of these molecules has been closely correlated with the survival and progression of cancers, including PEL [29,30]. Together, these results suggest that 5-AZA could be a promising strategy to increase the sensitivity of PEL cells to treatment with STAT3 tyrosine inhibitors.

### 3.3. 5-AZA/AG490 Combination Strongly Impairs the Release of Pro-Survival Cytokines by PEL Cells

**Figure 3:**
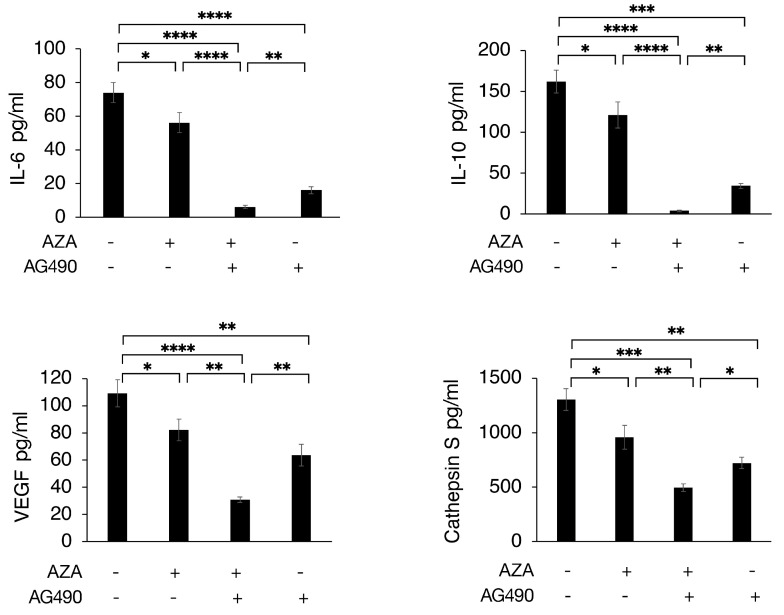
Effect of AG490, 5-AZA, and their combination on cytokine and cathepsin S release. The BC3 cell line was treated by AG490, 5-AZA or with 5-AZA and AG490 combination. The amount in pg/mL of IL-6, IL-10, VEGF and cathepsin S, as evaluated by Luminex, is shown. Means ± standard deviation (SD) of three independent experiments are shown. *p*-values: * < 0.05; ** < 0.01; *** < 0.001; and **** < 0.0001.

One of the effects correlated with STAT3 activation is the release of cytokines, including IL-6 and IL-10, reported to play a role in the survival of PEL cells [31]. Here, we found that 5-AZA further impaired IL-6, IL-10 and VEGF release compared to AG490 (Figure 3). This effect is particularly important, as apart from sustaining PEL cell survival and growth, these cytokines play a role in creating an immune-suppressive environment that allows cancer cells to escape immune recognition. Finally, we found that the 5-AZA/AG490 combination strongly reduced the release of cathepsin S, a molecule shown to regulate the crosstalk between T cells and malignant B cells and to promote lymphomagenesis, thus representing a novel therapeutic target [32].

### 3.4. EZH1/2 Inhibitor Valemetostat (DS-3201) Slightly Influences PEL Cell Survival and STAT3 Activation

**Figure 4:**
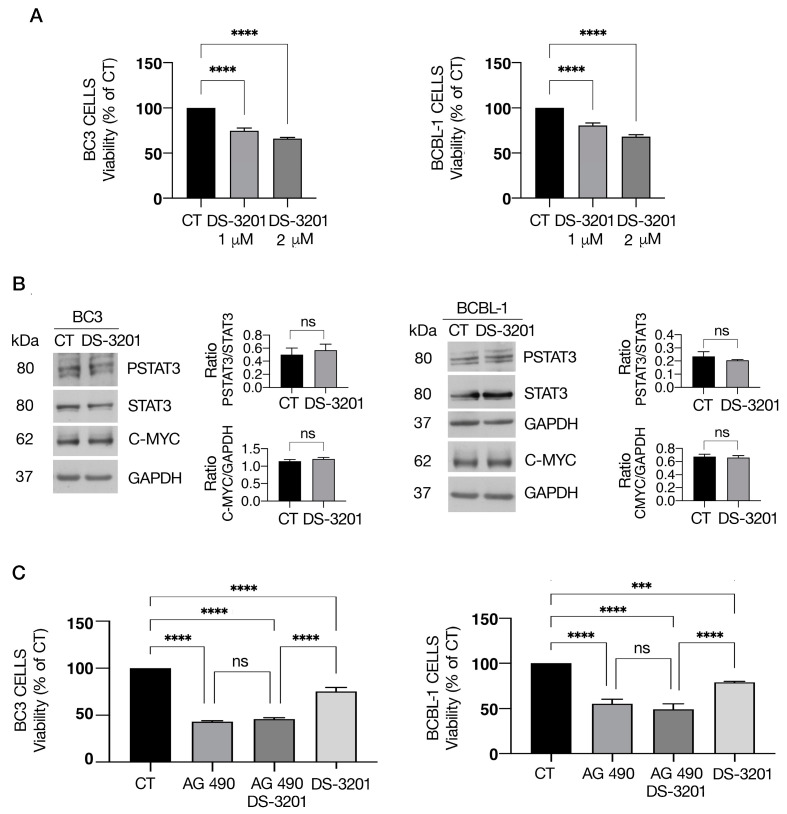
Valemetostat (DS-3201) induces mild cytotoxicity in PEL cells alone or in combination with AG490. BC3 and BCBL-1 cell lines were treated singly or in combination with valemetostat (DS-3201) and AG490. (**A**) Cell survival was estimated by trypan blue exclusion assay after treatment with valemetostat (DS-3201) (1–2 μM) for 48 h. The histograms represent the mean of the percentage of cell viability relative to the control plus S.D. (**B**) Protein expression level of pSTAT3, STAT3, and c-MYC, as evaluated by Western blot analysis. GAPDH was used as a loading control, and one representative experiment is shown. The histograms represent the densitometric analysis of specific proteins and the appropriate control from three experiments. Data are represented as means plus S.D. (**C**) Cell survival was estimated by trypan blue exclusion assay after treatment with valemetostat (DS-3201) (1 μM) and AG 490 (100 μM). The histograms represent the mean of the percentage of cell viability relative to the control plus S.D. *p*-value: *** < 0.001; and **** < 0.0001, ns means no significance.

STAT3 activity has been reported to be directly controlled by protein methylation mediated by EZH2 histone methyltransferase, which may lead to an increase in STAT3 activation [12]. We therefore evaluated whether the inhibition of EZH1/2 could affect PEL cell survival and reduce STAT3 tyrosine phosphorylation. As shown in Figure 4A, EZH1/2 inhibitor valemetostat (DS-3201), used at two different doses and reported to be effective against other cancers [33], slightly reduced PEL cell survival. Accordingly, at the highest dose used, it also slightly affected STAT3 phosphorylation as well as the expression of c-Myc (Figure 4B). Finally, we found that DS-3201 in combination with AG490 slightly influenced the cytotoxic effect of this drug against PEL cells (Figure 4C).

## 4. Discussion

Epigenetic modifications, including DNA methylation and histone modifications, have been reported to play a key role in driving carcinogenesis, alongside genetic mutations [34]. Interestingly, in some cases, epigenetic changes may predispose individuals to the occurrence of mutations and interestingly, mutations may affect the genes able to induce epigenetic changes [35]. Regarding STAT3, a transcription factor strongly involved in carcinogenesis [36], its activation may be regulated by DNA methylation of SOCS3 and PTPN6 promoters or by the methylation of the STAT3 protein itself, which is mediated by histone methylation enzymes such as EZH1/2. Methylation of STAT3 may increase its activation, as for example in the case of AML, in which STAT3 methylation influenced the outcome of a treatment targeting STAT3 phosphorylation [19]. In this study, we explored the possibility of using a demethylating agent to target STAT3 in PEL cells for the first time, as inhibition of STAT3 represents one of the few promising opportunities to treat this aggressive lymphoma [6]. Treatment with 5-AZA has been reported to increase overall survival in patients with higher-risk myelodysplastic syndromes compare to conventional care [37] and to induce a sustained response in angioimmunoblastic T-cell lymphoma patients [38]. Regarding adverse effects, 5-AZA has been shown to induce myelosuppression, including neutropenia, thrombocytopenia, and anemia, although these effects decreased in responding patients as therapy continued [39]. In the present study, we found that treatment of PEL cells with the DNA demethylating agent 5-AZA reduced STAT3 phosphorylation and impaired PEL cell survival, suggesting that it may be promising against PEL patients, while the inhibition of EZH1/2 histone-methylating enzyme by DS-3201 (valemetostat) slightly affected either STAT3 activation or survival in PEL cells. 

Furthermore, we found that 5-AZA treatment enhanced the cytotoxic effect of the JAK2/STAT3 pathway inhibitor AG490 against PEL cells. Indeed, although this drug is able to reduce the constitutive activation of STAT3, some cells still resist the treatment; thus, 5-AZA could help to potentiate the cytotoxic effects mediated by AG490. At the molecular level, 5-AZA increased the expression of SOCS3, known to be among the numerous targets of STAT3 and known to be able to negatively regulate its activation, suggesting that methylation of the SOCS3 promoter could contribute to STAT3 activation in PEL cells, as previously reported in bladder cancer cells in which hypermethylation of SOCS3 represents a prognostic biomarker [40]. Alongside STAT3, SOCS3 may interact with several other signaling pathways, affecting cancer diagnosis and prognosis [41]. SOCS3 silencing has been reported to increase the expression of c-Myc and tumorigenesis [42], and accordingly, its expression has been found to be lower in breast, ovarian, and colon cancers [43] and glioblastoma [44] in comparison with tumor-adjacent tissues.

Methylation of SOCS1, another member of the SOCS family, has been shown to be hypermethylated and contribute to STAT3 activation in MM [45], a cancer that shares several similarities with PEL [46]. Moreover, here, we found that the expression of PTPN6/SHP1 tyrosine phosphatase increased following 5-AZA treatment, suggesting that DNA methylation of this gene may also contribute to the activation of STAT3 in PEL cells. Interestingly, SHP1 methylation and JAK/STAT epigenetic modifications have been shown to play a role in the pathogenesis of multiple myeloma [20]. The upregulation of SOCS3 and PTPN6 by 5-AZA treatment, in addition to a stronger STAT3 inhibition in combination with AG490, led to a more intense downregulation of pro-survival molecules regulated by this transcription factor, such as c-Myc, Cyclin D1 and HSP27. It also contributed to a further reduction in pro-tumorigenic cytokine and cathepsin S secretion, with important implications for tumors escaping immune recognition. The study of epigenetic modifications of DNA as well as of histones and the presence of mutations of the enzymes inducing epigenetic modifications in different type of lymphoma, including peripheral T-Cell lymphomas, is essential to our understanding of how the epigenetic landscape influences gene expression, metabolism, and drug resistance [47,48].

## 5. Conclusions

In conclusion, the results obtained in this study suggest that the use of 5-AZA could upregulate the expression of STAT3 inhibitors such as SOCS3 and PTPN6/SHP1, reduce the activation of STAT3 and the expression of its pro-survival targets, and impair the survival of PEL cells. Notably, 5-AZA in combination with AG490 tyrosine kinase inhibitor increased the sensitivity of PEL cells to the treatment and further counteracted the release of pro-tumorigenic and immunosuppressive factors [49], making the tumor microenvironment more unfavorable for tumor cells.

## Data Availability

The datasets generated and analyzed during the current study are available from the corresponding author upon reasonable request.
